# Artificial intelligence in endodontic decision-making: hallucinations and emerging challenges for clinical practice

**DOI:** 10.2340/aos.v85.46515

**Published:** 2026-07-16

**Authors:** Carlos Wesley Lopes Brasil da Silva, Mariana Souza D’Afonseca, Ana Luiza Gonzaga Zonca, Jardel Francisco Mazzi Chaves, Fabiane Carneiro Lopes Olhê, Henrico Badaoui Strazzi-Sahyon, Gustavo Sivieri-Araujo

**Affiliations:** a Department of Restorative Dentistry and Endodontics, Discipline of Endodontics, Araçatuba School of Dentistry, São Paulo State University – UNESP, Araçatuba, SP, Brazil; b Department of Restorative Dentistry, Discipline of Endodontics, Ribeirão Preto School of Dentistry, São Paulo University – USP, Avenue Café, Ribeirão Preto, SP, Brazil; c Department of Prosthodontics and Periodontology, Bauru School of Dentistry, University of São Paulo – USP, Bauru, SP, Brazil

Dear Editor,

The accelerated incorporation of Artificial Intelligence (AI) into contemporary Dentistry has profoundly reshaped the landscape of diagnostic reasoning, treatment planning, and clinical education. In particular, the emergence of large language models (LLMs) as accessible cognitive support tools has generated substantial interest regarding their potential role in assisting clinicians during complex decision-making processes. Within Endodontics, where diagnostic interpretation frequently requires the integration of imaging findings, symptomatology, and biological plausibility, the appeal of such systems is especially evident.

Recent investigations have explored the capacity of LLMs to answer endodontic questions, support educational workflows, and assist in clinical reasoning tasks, suggesting a potentially expanding role in evidence-informed decision support [1]. Recent evidence has further demonstrated that AI-powered chatbots can provide consistent and scientifically reliable responses in endodontic practice, reinforcing their potential as adjunctive tools while simultaneously highlighting the need for critical professional appraisal of their outputs [2]. As these systems become progressively embedded into academic and clinical environments, their influence on diagnostic and therapeutic judgment is expected to increase substantially.

Among the most critical challenges in Endodontic practice is the indication for nonsurgical retreatment, a decision that demands a highly refined interpretation of clinical, radiographic, and cone-beam computed tomography findings. The assessment of persistent apical pathology, missed canals, obturation deficiencies, coronal leakage, untreated isthmuses, and complex accessory anatomy requires not only technical expertise but also advanced contextual reasoning. An inaccurate decision at this stage may lead either to unnecessary retreatment, with avoidable biological and financial burden, or to failure in intervening when pathological progression demands immediate care. In this context, AI-based systems may emerge as promising adjunctive tools to reduce uncertainty and support diagnostic standardization [1, 3].

However, the growing enthusiasm surrounding LLM-assisted decision-making must be tempered by critical scientific scrutiny. Although recent investigations have demonstrated satisfactory accuracy and consistency of AI-powered chatbots in answering endodontic questions [2], one of their most concerning limitations remains the propensity to generate factually incorrect yet linguistically persuasive outputs, a phenomenon widely recognized as AI hallucination [4]. Within the clinical context, hallucinations may manifest as fabricated scientific references, unsupported diagnostic pathways, distorted interpretation of radiographic findings, or plausible-sounding treatment recommendations devoid of evidence-based substantiation.

This limitation becomes particularly critical in Endodontics, where diagnostic conclusions often depend on subtle radiographic and tomographic cues that demand expert interpretation. The presence of fragmented clinical information, incomplete symptom history, ambiguous imaging findings, or poorly structured prompts may further amplify the risk of erroneous outputs. Recent studies have demonstrated that LLMs used for medical decision support are particularly vulnerable to hallucination when case information is incomplete, conflicting, or contextually ambiguous [5].

Such vulnerability assumes even greater significance in Endodontics, where the identification of unobturated canals, isthmuses, accessory anatomy, vertical root fractures, external resorptive defects, and persistent periapical lesions frequently requires multimodal interpretation and expert biological reasoning. In these scenarios, hallucination-driven outputs may not merely represent computational inaccuracies but may directly translate into clinically consequential diagnostic errors.

Moreover, emerging evidence from multimodal assurance analyses indicates that LLM-based decision support systems are highly vulnerable to adversarial hallucination attacks, raising important concerns about the trustworthiness of AI-generated diagnostic recommendations in real-world clinical settings, where patient information is often incomplete, heterogeneous, and context dependent [5]. This issue is particularly relevant in Dentistry, where diagnostic reasoning is inherently dependent on the convergence of subjective symptoms, clinical findings, and imaging interpretation.

Additionally, the opacity of algorithmic reasoning in LLM-based systems may further limit clinician trust and hinder traceable diagnostic justification, particularly in complex endodontic cases where treatment decisions must be biologically and radiographically supported. The inability to clearly explain how a recommendation was generated may compromise both clinical confidence and the reproducibility of diagnostic reasoning [5–7].

Beyond diagnostic reliability, the integration of LLM-based recommendations into endodontic practice also raises important ethical and medico-legal concerns. In situations where AI-generated outputs contribute to an incorrect retreatment decision, questions regarding professional accountability, informed consent, and standard-of-care compliance become increasingly relevant. This dimension further reinforces that such systems must remain under strict clinician supervision [8].

Beyond direct clinical implications, the uncritical use of LLMs may also shape diagnostic reasoning patterns among undergraduate students, residents, and early-career clinicians. In Endodontic education, excessive reliance on AI-generated recommendations without adequate critical appraisal may inadvertently weaken the development of independent diagnostic reasoning, radiographic interpretation skills, and biologically grounded clinical judgment [4, 5, 8].

Although AI and LLMs have the potential to enhance endodontic diagnosis, treatment planning, and educational support, their integration into clinical practice should remain strictly adjunctive and continuously guided by expert professional judgment [1, 5, 6]. Until robust validation frameworks, factual verification protocols, and clinically tested multimodal models become widely available, the indiscriminate adoption of these technologies may compromise diagnostic accuracy, weaken clinical accountability, and ultimately expose patients to inappropriate therapeutic decisions [8]. Therefore, the future of AI in Endodontics should be defined not solely by technological sophistication but by its ability to provide transparent, reliable, and biologically sound support that enhances diagnostic safety and strengthens responsible clinical decision-making [5, 7, 9].
